# Cone-Beam Computed Tomography Assessment of Root Canal Transportation by Neoniti and Reciproc Single-File Systems

**DOI:** 10.7508/iej.2016.02.004

**Published:** 2016-03-20

**Authors:** Fariborz Moazzami, Leila Khojastepour, Mohammadreza Nabavizadeh, Mina Seied Habashi

**Affiliations:** a *Department of Endodontics, Dental School, Shiraz University of Medical Sciences, Shiraz, Iran; *; b *Department of Oral and Maxillofacial Radiology, Dental School, Shiraz University of Medical Sciences, Shiraz, Iran; *; c *Prevention of Oral and Dental Diseases Research Center, Dental School, Shiraz University of Medical Sciences, Shiraz, Iran*

**Keywords:** Canal Transportation, Cone-Beam Computed Tomography, Neoniti, Reciproc, Root Canal Preparation

## Abstract

**Introduction:**

The aim of this *in vitro *study was to compare the canal transportation of two single-file engine-driven systems, Neoniti and Reciproc, using cone-beam computed tomography (CBCT).

**Methods and Materials:**

Forty-five non-calcified roots with mature apices and apical curvature of 15-30 degrees were selected from extracted human maxillary molars for this study. Samples were randomly divided into two groups (*n*=20) and a control group (*n*=5) and canal preparation with either system was performed according to manufacturers' instructions. Pre- and post-instrumentation CBCT images were captured and the amount of canal transportation within the files was calculated at levels of 3, 4, and 5 mm from the apex. The independent sample t-test was used to analyze the statistical significance between the two groups. The level of significance was defined at 0.05.

**Results:**

Reciproc created more canal transportation compared to Neoniti in both mesiodistal and buccolingual directions. The difference between the two systems was statistically significant in all evaluated distances from the apex (*P*<0.001). During this study fracture of one file (25/0.08) in the Neoniti group occurred.

**Conclusion:**

Neoniti and Reciproc systems have significant difference in terms of creating canal transportation. Reciproc created more canal transportation in buccolingual and mesiodistal dimensions.

## Introduction

Cleaning and shaping of the root canal system should eliminate or at least reduce the intra-canal micro-organisms and also maintain the original shape of the root canal. Transportation is one of the mishaps that deviate canal terminus to a new location which can jeopardize the treatment outcome [1, 2]. Eventually, apical transportation may lead to zipping or perforation of the canal [3]. With the introduction of nickel-titanium (NiTi) rotary systems into endodontic practice, the chances of procedural errors such as canal transportation, zip, ledge and striping perforation have decreased [4-7].

New systems with the reduced number of files and even single-file systems have innovated endodontic practice. Several studies were designed to compare the preparation ability of single-file systems in severely curved root canals [8-11]. Bane *et al*. [12] compared the shaping ability of two single-instrument systems with ProTaper as a conventional rotary system. They found that WaveOne and Reciproc prepared curved canals faster and with less procedural accidents.

A recent comparative study by Saber *et al. *[13] has shown that the use of WaveOne and Reciproc instruments resulted in significantly less canal transportation than OneShape instrument. They attributed these finding to M-Wire alloy in WaveOne and Reciproc and also to their reciprocal motion.

**Figure1:**
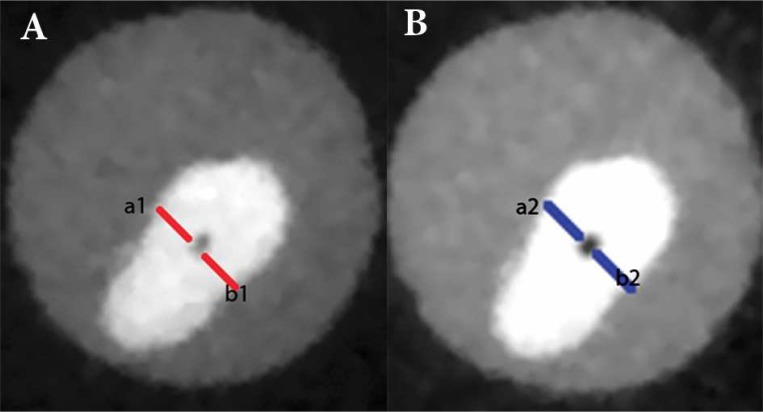
CBCT images at 5-mm distance from the apex; *A)* pre- and *B)* post-instrumentation images with Neoniti. Amount of canal transportation was obtained from (*a*_1_*-a*_2_)-(*b*_1_*-b*_2_) formula

Single-file rotary systems may be classified on the basis of their motion into rotating and reciprocating files. Neoniti A1 (NEOLIX, Châtres-la-Forêt, France) is one of these newly introduced single-file systems with full rotary motion. This system has continuous rotating movement and is made up of special alloy that permits the file flexibility. This system is produced with three different sizes (20/0.08, 25/0.08 and 40/0.08) that are recommended to be used with speed of 300 to 500 rpm and torque limit of 1.5 N/cm. According to the manufacturer this file offers many advantages such as sharp cutting edges, single-file technique, Gothic-like tip design and built-in abrasive properties [14].

Reciproc (VDW, Munich, Germany) has S-shaped cross-section, a non-cutting tip and sharp cutting edges that shapes the canal by means of a reciprocal back-and-forward motion (150 degrees counterclockwise and then 30 degrees clockwise). This single file-system is available at three different sizes and tapers; R25 (25/0.08), R40 (40/0.06) and R50 (50/0.05) [12]. Previous studies using this system in extracted teeth have shown that it can maintain the original shape of root canal similar to conventional rotary systems [8, 15]. Reciproc files have a continuous taper over the first 3 mm of their working part followed by a decreasing taper until the shaft [16].

Previous studies that have compared systems of root canal shaping with continuous rotation and reciprocating motion, reported less canal transport and more root canal centrality with reciprocating motion [17, 18]. On the contrary, some studies stated that reciprocating movement caused more transportation [10, 19]. So still there is limited information regarding the influence of reciprocating motion on canal transportation compared to continuous rotation.

The aim of this experimental *in vitro* study was to compare canal transportation after preparation of root canals using either Neoniti or Reciproc single-file systems by using cone-beam computed tomography (CBCT).

**Figure2:**
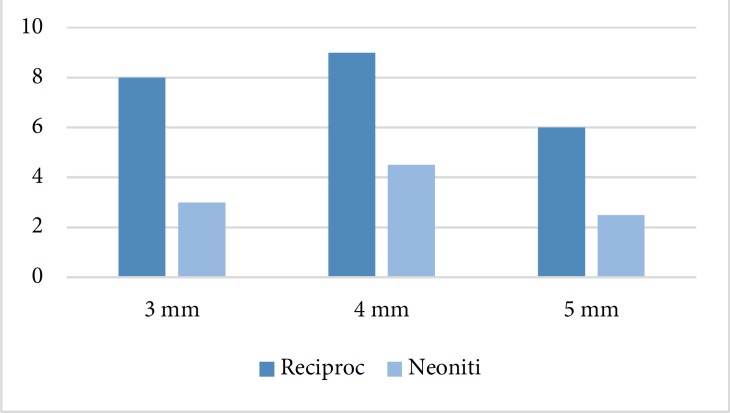
The rate of transportation at 3, 4 and 5 mm from the apex in Neoniti and Reciproc systems

## Materials and Methods

This study was approved by the ethics committee of Shiraz University of Medical Sciences (Grant No.: 8769) and was done on 45 single-canal mesiobuccal roots of human maxillary molars which were extracted for periodontal and prosthodontics problems. The canals had at least 19 mm working length and 15^°^-30^º^ apical curvature (according to Schneider’s method) [20]. The roots were checked radiographically to have mature apices and non-calcified canals [20, 21].

The teeth were disinfected with 5.25% sodium hypochlorite before the experiment. Access cavities were prepared with #4 high speed round carbide bur (Dentsply, Maillefer, Ballaigues, Switzerland) and the root canals were negotiated by using #10 K-file (Dentsply, Maillefer, Ballaigues, Switzerland). Roots without achievable patency with a #10 K-file to the major foramen, were excluded. Moreover, roots that did not allow passive placement of a #15 K-file within 1 mm of the apical foramen and root canals wider than size 20 at the apex were also discarded.

The working length was defined as the distance from the occlusal reference point to 0.5 mm short of the file length after extrusion of the #10 K-file through the apical foramen. All specimens were standardized at 19 mm working length by cutting off the crowns. 

For easier placement of teeth, the palatal and distobuccal roots were amputated at the apical furcation level and placed in 6×6 cm acrylic model. The teeth were mounted parallel to the walls of acrylic mold. Five roots were considered as control samples. The remaining teeth were randomly divided into two groups (*n=*20). These two groups were homogenized regarding the angle of curvature with unpaired t-test before the beginning of experiment.

Group one (*n*=20) were prepared with Reciproc file system (25/0.08) according to the manufacturer’s instructions, installed on a handpiece powered by electric torque control motor (Silver, VDW, Munich, Germany) set on the “Reciproc All” program.

In group two (*n*=20) root canals were prepared by using 25/0.08 Neoniti A1 (NEOLIX, Châtres-la-Forêt, France), powered by similar electric torque control motor with speed of 300 rpm and torque of 1.5 N/cm, according to manufacturer’s instructions. After every in-and-out movement, the file was pulled out for cleaning its flutes and canal irrigation was done with 2 mL of 2.5% normal saline using a 27-gauge irrigation needle. The files were used in the same manner until it reached the working length. One endodontist prepared all root canals in both groups and files were discarded after preparation of four canals.

Roots were scanned before and after preparation with CBCT device (NewTom VGi, QR SRL Co., Verona, Italy) with the following setup: 110 kVp, 9.5 mA and 0.1×0.1×0.1 mm voxel size and 0.100 mm axial thickness [22, 23].

The amount of apical transportation was compared between two groups in 3, 4 and 5-mm distances from the radiographic apex by using axial cross sections in NNT Viewer software (NNT software corporation, Yokohama, Japan) and the measurements were done with Adobe Photoshop CS5 (Adobe systems Inc., San Jose, CA, US) [23].

The degree of root canal changes was recorded separately in buccolingual and mesiodistal dimensions by Adobe Photoshop CS5 using the following formula (*a*_1_-*a*_2_)-(*b*_1_-*b*_2_), where *a*_1_ is the shortest distance between mesial (or lingual) aspect of non-instrumented canal to mesial (or lingual) edge of the root, and *a*_2_ is the shortest distance between mesial (or lingual) aspect of instrumented canal to the mesial (or lingual) edge of the root. Likewise *b*_1_ is the shortest distance between distal (or buccal) aspect of non-instrumented canal to distal (or buccal) edge of the root, and *b*_2_ is the shortest distance between distal (or buccal) aspect of instrumented canal to distal (or buccal) edge of the root [24] (Figure 1). The result 0 indicates no canal transportation, negative results means distal (or buccal) transportation and positive results show mesial (or lingual) transportation.

Independent sample t-test was used to compare the groups in both buccolingual and mesiodistal dimensions. SPSS software (SPSS version 17.0, SPSS, Chicago, IL, USA) was used for statistical analysis and the level of significance was set at 0.05.

## Results

The mean±SD for the angle of curvature of two groups using student’s t-test are shown in Table 1. During this study, there was one file fracture in the Neoniti group. This sample was substituted with another tooth in order to maintain sample volume. The mean±SD for mesiodistal and buccolingual transportation values in both systems are shown in Table 2. According to the mean degree of transportation in each section, Reciproc caused more transportation compared to Neoniti in both mesiodistal and buccolingual directions.

The difference between the two systems was statistically significant in all 3, 4 and 5 mm distances from the apex (Figure 2).

In both groups at 3 and 5 mm levels, buccolingual transportation was significantly more than mesiodistal transportation. However, at 4 mm from the apex mesiodistal transportation in Reciproc system was significantly more than buccolingual transportation. In Neoniti group buccolingual transportation was significantly more than mesiodistal transportation at 4-mm distances (*P*<0.001).

## Discussion

This experimental study investigated the amount of transportation induced by two engine-driven single-file systems, Reciproc and Neoniti, on extracted teeth using CBCT. It was found that Reciproc system produced more transportation compared to Neoniti in both mesiodistal and buccolingual directions at all examined sections and with similar apical preparation diameter.

The findings of this experimental study are consistent with those of Nabavizadeh *et al*. [10] in which Reciproc with reciprocating motion showed significantly higher transportation than BioRace system with rotational movements. 

On the contrary according to the investigation by Burklein *et al*. [8] Reciproc and WaveOne maintained the original canal curvature with no significant differences between these two reciprocating file systems with Mtwo and ProTaper. In another study that compared Reciproc and Twisted file systems, twisted file produced more transportation compared to Reciproc in both mesiodistal and buccolingual directions. However, the difference between the two systems was only statistically significant in the 5-mm distance from the apex [11].

**Table 1:** Mean (SD) of curvature of root canals

Groups (N)	Curvature (degrees)	Min	Max
**Reciproc (20)**	22.39 (2.45)	15	30
**Neoniti (20)**	23.24 (2.70)	15	30
*P-*value	**0.303**		

**Table 2:** Mean (SD) of transportation in mm at the defined levels (MD=mesiodistal, BL=buccolingual

**Group**	**Distance **	**MD transportation**	**BL transportation **	***P*** **-value**
**Neoniti**	**3 mm**	0.03 (0.0084)	0.04 (0.0076)	<0.001
**Reciproc**		0.08 (0.0091)	0.09 (0.0085)	<0.001
**Neoniti**	**4 mm**	0.04 (0.0076)	0.05 (0.0093)	<0.001
**Reciproc**		0.09 (0.0085)	0.07 (0.0075)	<0.001
**Neoniti**	**5 mm**	0.02 (0.023)	0.06 (0.0084)	<0.001
**Reciproc**		0.06 (0.008)	0.12 (0.008)	<0.001

More canal transportation with Reciproc may be due to its stiffness and the cross-sectional design of the instruments. Reciproc has a sharp double-cutting edge and S-shaped geometry, while Neoniti files have non-homothetic rectangular cross sections with rounded Gothic tips [19, 25]. Furthermore, Neoniti system does not have the usual metallic memory and tendency to rapidly return to straight position, the properties that may explain more centering ability of this rotary system. The manufacturer claimed that this special feature is due to the use of a newly developed wire-cut electrical discharge machining (EDM) process and an appropriate heat treatment which caused the special progressive flexibility of the files [26].

CBCT imaging is one of the newest methods for evaluation of root canal preparation techniques. The advantages of this system are low radiation dose and limited field of view that can improve resolution and produce more diagnostic abilities [27, 28]. In the present study the CBCT device was set at resolution of 0.1 μm, that is more accurate for determining the smallest alterations in root canal system compared to the previous studies [29-31].

The evaluation of transportation on resin block instead of extracted human teeth has some shortcomings. First, the hardness of dentin is more than resin, so for the cutting of dentin more force should be applied. Also, resin chips can obliterate the canal and hinder deep penetration of instruments [32].

Apical transportations more than 0.3 mm can lead to loss of seal in apical area and endanger treatment prognosis. In this experiment, there are significant differences in canal transportation of two groups, but the range of transportation was between 0.03-0.12 mm, that could not jeopardize the apical seal [33]. Although in Neoniti group one case of broken instrument occurred, it did not happen in Reciproc group and this can be attributed to the special reciprocating movement and S-shaped cross section of this system; therefore, using this system seems to be safer regarding file breakage.

## Conclusion

All instruments were clinically safe to use. Reciproc causes more canal transportation in buccolingual and mesiodistal dimensions.
